# Health-Related Quality of Life and Mobility Levels in ICU Survivors with Heel Pressure Ulcer: An Observational Study

**DOI:** 10.3390/nursrep16010030

**Published:** 2026-01-17

**Authors:** Filippo Binda, Federica Marelli, Veronica Rossi, Lucia Villa, Andrea Cislaghi, Giacomo Grasselli

**Affiliations:** 1 Department of Healthcare Professions, Fondazione IRCCS Ca’ Granda Ospedale Maggiore Policlinico, Via Francesco Sforza 35, 20122 Milan, Italy; federica.marelli@policlinico.mi.it; 2 Department of Anesthesia, Intensive Care and Emergency, Fondazione IRCCS Ca’ Granda Ospedale Maggiore Policlinico, Via Francesco Sforza 35, 20122 Milan, Italy; veronica.rossi@policlinico.mi.it (V.R.); lucia.villa@policlinico.mi.it (L.V.); andrea.cislaghi@policlinico.mi.it (A.C.); giacomo.grasselli@unimi.it (G.G.); 3 Department of Pathophysiology and Transplantation, University of Milan, Via Francesco Sforza 35, 20122 Milan, Italy

**Keywords:** pressure ulcer, intensive care units, quality of life, mobility limitation

## Abstract

**Background/Objectives**: Heel pressure ulcers are a relevant complication in critically ill patients and may negatively affect recovery after ICU discharge. This study investigated health-related quality of life (HRQoL) and mobility levels one year after ICU discharge in survivors who developed heel pressure ulcers. **Methods**: A prospective observational study was conducted in the ICU of an academic tertiary-level hospital in Milan (Italy) from 1 January 2023 to 31 December 2024. Adult survivors were enrolled, and HRQoL was assessed using the EQ-5D-5L questionnaire. Functional status at ICU discharge was evaluated using the Manchester Mobility Score and Barthel Index. This study adhered to the Strengthening the Reporting of Observational Studies in Epidemiology (STROBE) guidelines. **Results**: Among 3144 ICU admissions, 52 survivors were enrolled. At ICU discharge, functional status was markedly impaired: only 15 patients (28.9%) were able to stand upright according to the Manchester Mobility Score, and none achieved even moderate levels of independence. At one year, 47 patients (90.4%) completed the follow-up, and 15 of them (31.9%) continued to report moderate-to-severe mobility limitations. The mean EQ-5D index value was 0.75 (SD 0.27), representing a significant reduction compared with Italian population norms (*p* < 0.001). **Conclusions**: ICU survivors who developed heel pressure ulcers exhibit reduced HRQoL at one year after discharge. These findings emphasize the need for structured post-ICU rehabilitation and targeted follow-up.

## 1. Introduction

Advances in critical care and population aging have produced a growing cohort of critical-illness survivors, while short-term mortality within the intensive care unit (ICU) continues to fall [1]. After ICU discharge, many survivors experience a reduced physical and psychological health as well as impaired social functioning [2]. These outcomes are closely inter-related: functional disability is consistently associated with poorer health-related quality of life (HRQoL) [3]. Poor HRQoL among ICU survivors is a significant concern linked to higher mortality, financial burden, and family caregiver burden [4]. One of the major contributors to such disability is the development of pressure ulcers during the ICU stay, particularly in anatomically vulnerable areas [5]. The impact of pressure ulcers on reducing HRQoL is substantial, causing pain and discomfort and affecting rehabilitation, mobility, and psychological, physical and social aspects of people lives [6,7].

The occurrence of pressure ulcers in patients admitted to ICU represents an important concern, given their high prevalence and the risks related to patient safety [8]. Notably, the prevalence of pressure ulcers among patients in ICUs remains high: a global point prevalence study conducted across 1117 ICUs in 90 countries reported a pressure ulcer prevalence of 26.6% (6747 out of 13,254 patients; 95%CI: 25.9–27.3) [9]. Among the reported pressure ulcers, 59.2% (n = 3997) were acquired after the ICU admission [9]. The heel is the second most common anatomic site for pressure ulcers due to its limited soft tissue padding and frequent contact with the bed surface, friction, and shear forces [10,11].

Heel pressure ulcers are also associated with significant pain and functional impairment, resulting in reduced mobility [12]. From a biomechanical perspective, these lesions directly compromise the initial contact and loading-response phases of gait, during which the calcaneus normally absorbs impact and stabilizes the ankle. Pain and tissue damage disrupt this mechanism, prompting patients to adopt antalgic gait strategies that further limit walking capacity [13,14]. Importantly, the risk of developing heel pressure ulcers is strongly influenced by patient-specific factors that also predispose to poor functional recovery [15]. Advanced age, inadequate nutritional status, prolonged immobility, and obesity increase sustained heel loading and reduce tissue tolerance to pressure [15]. In parallel, chronic conditions such as diabetes mellitus, end-stage renal disease, and peripheral vascular disease impair microvascular perfusion, sensory feedback, and wound healing, thereby exacerbating tissue damage and prolonging functional limitations [16,17]. These combined mechanisms contribute not only to delayed mobilization but also to longer hospital stays and they negatively affect the overall HRQoL of hospitalized patients [18].

HRQoL is a multidimensional and dynamic [19] and includes different components, such as physical health, level of independence, psychological well-being, and social relationships [20]. Several instruments have been developed to measure HRQoL and have been used in survivors of critical illness [21]. In particular, many studies have specifically reported the use of the EQ-5D (EuroQol 5-Dimension) to assess HRQoL in patients discharged from ICU [22,23]. Moreover, it has been shown to be particularly useful in vulnerable populations, as it more effectively captures clinical differences based on disease type and severity [24,25].

Despite growing attention to heel pressure ulcer prevention in critical care settings, less is known about their long-term consequences, especially after ICU discharge [26]. ICU-acquired heel pressure ulcers may persist or worsen post-discharge [27], delaying wound healing and impairing functional recovery. A recent systematic review has reported an association between pressure ulcers and reduced HRQoL in patients recruited from acute care settings [28]; however, the functional consequences of heel pressure ulcers among ICU survivors remain insufficiently explored. In particular, there is limited evidence regarding their impact on post-ICU functional recovery, including mobility trajectories, walking capacity, and independence in activities of daily living (ADL). Clarifying these functional outcomes is crucial to support individualized rehabilitation pathways, improve discharge planning, and develop structured and outcome-oriented ICU follow-up programs. Therefore, the aim of this study was to investigate HRQoL and mobility levels one year after ICU discharge in survivors with heel pressure ulcers.

## 2. Materials and Methods

### 2.1. Study Design and Setting

This prospective observational study was conducted at the Fondazione IRCCS Ca’ Granda Ospedale Maggiore Policlinico, a tertiary-level academic hospital in Milan (Italy), between 1 January 2023 and 31 December 2024. The Department of Anesthesia, Intensive Care, and Emergency comprises 24 beds and serves as an ECMO referral center.

The study population included all adult patients admitted to the ICU who developed a heel pressure ulcer on one or both heels during their ICU stay or had a pre-existing heel pressure ulcer at the time of ICU admission. Only patients who had been discharged alive from the ICU were included, regardless of the healing status of the heel pressure ulcer. Patients with a documented diagnosis of cognitive impairment or those with communication difficulties that precluded reliable self-reporting were also excluded. Likewise, patients who died during hospitalization, did not provide informed consent, or declined to participate in the follow-up interview were excluded. The study was approved (approval number 765_2022) by the local Ethics Committee (Comitato Etico Milano area 2). Informed consent was obtained from all adult patients included in this study.

Because heel pressure ulcers are relatively uncommon, we prospectively enrolled every consecutive eligible patient who survived to ICU discharge during a recruitment period of 24 months. To detect a 0.15-point deviation from population norms with a power of 90%, even after accounting for an anticipated 10% attrition rate, we planned to enroll at least 52 people surviving the ICU with heel pressure ulcers. A sample with this size yields a half-width 95% confidence interval of the EQ-5D index value of 0.076 (below the 0.08 minimal clinically important difference for ICU survivors reported) [29].

### 2.2. Data Collection

Data for this prospective observational study were obtained from electronic medical records and from patient follow-up interviews conducted one year after ICU discharge. All data were recorded using the REDCap electronic data capture tool (version 14.3.13) [30]. Sociodemographic variables included age, sex, body mass index (BMI), presence of a caregiver, and employment status. Additional clinical data were collected, including comorbidities, the use of medical devices, type of extracorporeal support, duration of mechanical ventilation (MV), ICU length of stay, and discharge setting. Heel pressure ulcers were assessed according to the National Pressure Ulcer Advisory Panel (NPUAP) criteria [31]. Staging was documented at ICU admission for patients presenting with pre-existing heel pressure ulcers, whereas for ulcers acquired during the ICU stay, staging was performed at the time of onset. In all cases, heel skin integrity was inspected daily as part of routine nursing care, with assessments carried out by bedside clinicians. The risk of pressure ulcer onset was evaluated using the Braden Scale, which considers factors such as sensory perception, skin moisture, activity, mobility, nutrition, and the presence of friction and shear [32].

Mobility levels were described using the Manchester Mobility Score, a seven-point scale validated for assessing mobility in critical care settings [33]. To define patients’ levels of autonomy prior to ICU discharge, the Barthel Index was used to measure functional independence in ADL [34]. The study results were reported following the Strengthening the Reporting of Observational Studies in Epidemiology (STROBE) guidelines [35].

### 2.3. Health-Related Quality of Life

The primary outcome was the assessment of HRQoL, measured using the EuroQol five-dimension, five-level questionnaire (EQ-5D-5L) [36]. The questionnaire includes five dimensions: mobility, self-care, usual activities, pain/discomfort, and anxiety/depression—each with five levels: no problems (1), slight problems (2), moderate problems (3), severe problems (4), and extreme problems/unable to (5). To facilitate participant adherence and minimize loss to follow-up, the EQ-5D-5L questionnaire was administered via telephone interview conducted one year after ICU discharge. During the interview, patients were asked to indicate their current health status by selecting the most appropriate response in each dimension. These responses are coded as single-digit numbers representing the severity level for each domain and are combined into a five-digit code describing the individual’s overall health status. In addition, the EQ visual analogue scale (EQ-VAS) captures the patient’s self-rated health on a vertical scale ranging from 0 (the worst imaginable health status) to 100 (the best imaginable health status), reflecting their overall perception of health. Finally, the EQ-5D index value provides a single summary score representing the respondent’s health status, derived from the five-digit health state using a country-specific value set. The normative data for the Italian EQ-5D index value show a mean value of 0.93 (SD 0.11), with scores gradually decreasing with age [37]. For the administration of the EQ-5D-5L questionnaire, a regular non-commercial use license was obtained following registration on the EuroQol website (registration number: 63245).

### 2.4. Statistical Analysis

All statistical analyses were performed using Jamovi software, version 2.3.21 (Jamovi Project, 2025). Continuous variables were summarized as mean and standard deviation (SD) or median and interquartile range (IQR), depending on the distribution assessed using visual inspection and the Shapiro–Wilk test. Categorical variables were reported as absolute and relative frequencies. The primary outcome measure, HRQoL assessed using the EQ-5D index value, was analyzed descriptively and compared across subgroups using the Mann–Whitney U test. Associations between demographic or clinical variables and HRQoL were explored through univariate analyses. Additionally, EQ-5D index values were compared with age- and sex-specific Italian population normative values using one-sample *t*-tests, with each normative EQ-Index value applied as the test mean. A two-sided *p*-value of <0.05 was considered statistically significant.

## 3. Results

During the study period, a total of 3144 adult patients were admitted to the ICU, representing the broader critical care population of the hospital. Among these, 73 patients (2.3%) were identified as presenting with or developing heel pressure ulcers during their ICU stay. Of these, 21 died prior to hospital discharge. The final study cohort included 52 survivors with heel pressure ulcers.

Table 1 summarizes the sociodemographic and clinical characteristics of the cohort.

The mean age of the patients was 61.7 years (SD 16.4, range: 18–83 years), and the average body mass index (BMI) was 25.1 kg/m^2^ (SD 5.4). Regarding organ support and ICU treatments, 69.2% (36/52) of patients required invasive MV, with a median duration of 13.0 days (IQR 4.0–27.3). A statistically significant association was observed between length of MV and the development of heel pressure ulcers during the ICU stay (*p* = 0.008). Renal replacement therapy and extracorporeal membrane oxygenation (ECMO) were used in 21.2% (11/52) and 11.5% (6/52) of patients, respectively. Enteral nutrition via nasogastric tube was provided to 61.5% (32/52) of patients for a median duration of 12.0 days (IQR 3.5–26.5). The median ICU length of stay was 13.5 days (IQR 4.0–28.5) and total hospital stay was 38.5 days (IQR 26.8–72.0); patients spent a median of 17.0 days (IQR 9.0–29.0) on the ward after ICU discharge. Recovery of heel pressure ulcers occurred in only 13.4% (7/52) of patients before ICU discharge, whereas 36.5% (19/52) had unresolved lesions at hospital discharge, including 10 patients who were transferred to rehabilitation facilities.

The Braden Scale score indicated a high risk for pressure ulcers (≤12 points) in 96.1% (50/52) of patients. According to the NPUAP classification, of the 81 heel pressure ulcers, 44.4% (36/81) were classified as stage I, 38.3% (31/81) as stage II, and 17.3% (14/81) were unstageable. Additional pressure ulcers at other anatomical sites were observed in 76.9% (40/52), with the sacral region being the most affected for 14 patients (26.9%).

### Functional Status and Follow-Up

Functional status at ICU discharge was severely compromised, as shown in Table 2. Based on the Manchester Mobility Score, only 28.9% (15/52) of patients were able to bear weight on their feet and achieve an upright standing position. In line with these results, the Barthel Index also reflected substantial functional dependence, with no patients reaching levels of moderate or slight dependency.

At one year following ICU discharge, 90.4% (47/52) of patients were successfully contacted and provided informed consent to complete the EQ-5D-5L questionnaire. As shown in Table 3, 57.4% (27/47) of patients reported no problems with mobility, while 31.9% (15/47) experienced moderate-to-severe limitations (Levels 3–5), indicating a substantial degree of residual functional impairment. Investigating the mobility domain, other aspects of functional status were evaluated at follow-up: a total of 61.7% (29/47) reported being able to walk without the use of devices, although 68.1% (32/47) stated they were not engaging in any form of physical activity. Moreover, 59.6% (28/47) of patients had not resumed driving motor vehicles. The domains of mobility, self-care, and usual activities were the most frequently reported as Level ≥ 3, highlighting the impact of impaired mobility on ADL. Interestingly, almost 80% of ICU survivors reported Level ≤ 2 for the dimension of pain/discomfort.

**Figure 1:**
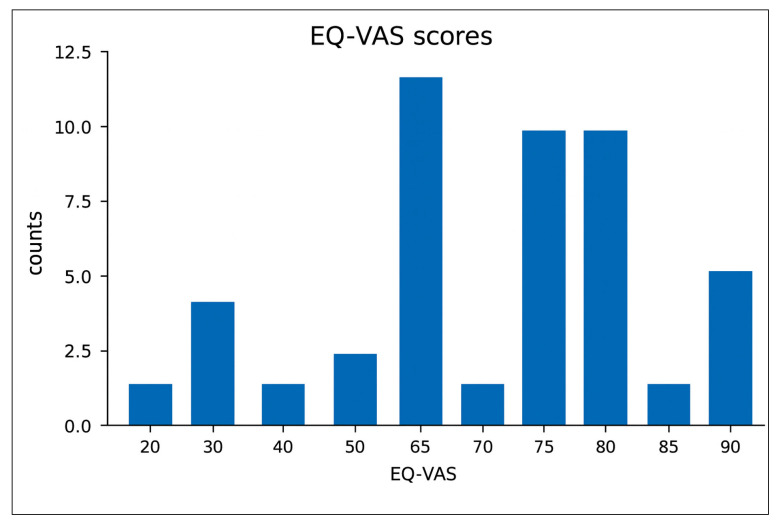
EQ-VAS.

EQ-VAS scores, as reported in Figure 1, ranged from 20.0 to 90.0, with a median of 70.0 (IQR 60.0–80.0) and mean of 65.9 (SD 17.6). These values reflect a moderate level of perceived health and well-being, with considerable variability across the cohort. The distribution of scores suggests that, while some ICU survivors with heel pressure ulcers report relatively favorable health status, a substantial proportion continue to experience notable limitations in their overall health perception one year after ICU discharge.

The EQ-5D index value ranged from −0.17 to 1.00, with a median of 0.82 (IQR 0.59–1.0) and a mean of 0.75 (SD 0.27), indicating a wide variability in perceived health status. As shown in Table 4, subgroup analyses revealed no statistically significant differences in EQ-5D index value scores by sex, age, or BMI. However, the need for invasive MV was associated with significantly lower EQ-5D index value scores at follow-up, suggesting a lasting negative impact of respiratory support on HRQoL. Although patients with an ICU length of stay ≥ 72 h or those discharged to facilities other than home had lower mean EQ-5D index values, these differences did not reach statistical significance.

A comparison between this cohort of patients and the Italian EQ-5D-5L population norms showed a consistent reduction in HRQoL across all age subgroups. Overall, patients reported a markedly lower EQ-5D index value compared with the normative data (0.74 versus 0.93), and this difference was statistically significant (*p* < 0.001). When stratified by age, the greatest decrement was observed in the 35–44-year group (mean difference −0.33). However, statistical significance was reached only in the 65–74-year group (mean difference −0.20; *p* = 0.041). The remaining age groups showed smaller, non-significant differences, with mean differences of −0.24 for patients aged 45–54 years and −0.09 for those aged 55–64 years. This pattern likely reflects both the clinical heterogeneity of the sample and the limited number of observations in some subgroups. Sex-specific analyses showed that males experienced a significantly greater impairment in EQ-5D index value relative to normative values (*p* < 0.001), whereas the reduction observed in females did not reach statistical significance. Taken together, these findings highlight the substantial and persistent burden of impaired HRQoL in this cohort and underscore the gap between post-hospital recovery trajectories and the expected health status of the general population.

## 4. Discussion

This prospective observational study provides evidence that, one year after ICU discharge, survivors who developed heel pressure ulcers continue to exhibit clinically relevant impairments in mobility and HRQoL. Approximately one-third of patients continued to experience moderate-to-severe mobility limitations at follow-up, and those requiring invasive MV had significantly poorer HRQoL.

The prevalence of patients with heel pressure ulcers during the ICU stay was 2.3%, which is lower than the rates reported in other studies, where prevalence ranged from 8.8% to 12.6% [38,39]. However, determining the true prevalence remains difficult due to substantial heterogeneity in study populations, designs, and methodological rigor. The risk of developing heel pressure ulcers is often underestimated, and early detection remains a significant clinical challenge, particularly in critically ill patients [40]. The presence of multiple comorbidities, especially vascular disease and diabetic neuropathy, further increases the risk and complicates timely identification [41]. In addition, many clinicians lack the ability to accurately distinguish diabetic foot ulcers, vascular-related ulcers, and pressure ulcers [42]. Pressure ulcer prevention is particularly critical in intensive care settings, where patients are frequently sedated, mechanically ventilated, or subjected to interventions that significantly restrict mobility and reduce the opportunities to perform routine skin evaluations [43]. The adoption of standardized, evidence-based protocols for the prevention and management of pressure ulcers is essential to ensure high-quality care [44]. In particular, the present findings underscore the necessity of systematic heel inspection and the application of preventive measures at this vulnerable anatomical site to reduce the risk of pressure ulcers and subsequent functional disability.

Heel pressure ulcers, like other pressure ulcers, are the result of multiple contributing factors [45]. Evidence suggests that risk factors associated with the development of heel pressure ulcers may differ from those of other anatomical sites due to the heel’s distinctive skin characteristics and positioning [46]. A recent systematic literature review identified eight key factors associated with heel pressure ulcer development: age, Braden subscales “friction and shear” and “mobility”, diabetes, vascular disease, malnutrition, mechanical ventilation, perfusion issues and surgery [47]. The factors listed are also observed in the population of this current study: in particular, length of invasive MV was significantly associated with heel pressure ulcer development, supporting earlier studies linking ventilatory support to impaired mobility and heightened pressure ulcer risk [48,49,50]. Additional factors, such as ECMO or prolonged renal replacement therapy, may further increase patient vulnerability and the risk of heel pressure ulcer development [51,52]. ECMO delivered through femoral cannulation restricts lower-limb mobility and promotes externally rotated lower limb postures. Combined with the difficulty of repositioning hemodynamically unstable patients or those requiring strict circuit protection, these constraints lead to sustained heel loading and tissue ischemia [53,54].

A notable finding of this study is that only 13.4% of heel pressure ulcers had healed before ICU discharge. Such delayed healing likely reflects the combined effects of tissue ischemia, impaired mobility, and the physiological stress of critical illness, all of which hinder wound repair [55]. The persistence of heel pressure ulcers after ICU discharge may further limit weight-bearing capacity and negatively influence mobility trajectories [56].

Long-term functional disability after ICU discharge is a well-recognized condition among ICU survivors [57,58]. Functional status in our cohort, evaluated using the Barthel Index and Manchester Mobility Score, indicated significant dependency at ICU discharge, consistent with previously reported findings [59,60,61]. This substantial level of dependency further supports evidence that pressure ulcers can adversely affect functional recovery following ICU discharge [62]. Notably, only 9.6% of patients were able to reach an active sitting position by the end of the ICU stay: this finding is clinically meaningful due to the strong association between the timing of early mobilization, independence in ADL, and favorable recovery trajectories [63,64]. Furthermore, organizational factors play a crucial role in mobilization practices within the ICU [65,66]. For example, a national survey of Japanese ICUs found that the presence of dedicated rehabilitation therapists was associated with higher rates of out-of-bed mobilization and significantly facilitated the implementation of early mobilization strategies [67].

One year after ICU discharge, survivors with heel pressure ulcers continued to report notable functional limitations and reduced HRQoL. Although more than half of patients indicated no mobility problems, a substantial minority reported moderate-to-severe restrictions, demonstrating that mobility impairment remains a persistent and clinically relevant issue in this patient population. These data are consistent with the concept of a prolonged recovery trajectory following critical illness, where initial immobility, muscle atrophy, and ICU-acquired weakness can lead to long-term deficits in physical function [68]. The results are aligned with the global evidence indicating that mobility disfunction represents one of the most disabling sequelae of ICU survival and is strongly associated with limitations in ADL [69,70]. Notably, most survivors experienced post-ICU cognitive and psychological impairments, as well as new or worsening dependencies in ADL (especially bathing, dressing, and walking), which frequently persisted for months to years following hospital discharge [71,72].

HRQoL one year after ICU discharge was substantially impaired in this cohort of ICU survivors. The median EQ-VAS score of 70 indicates only a moderate perception of health status, with considerable variability suggesting that many patients continue to experience physical and psychosocial limitations long after hospital discharge. These findings are supported by previous studies highlighting the persistent burden of disability in ICU survivors, often extending months to years beyond the acute episode of critical illness [73,74]. The EQ-5D index value further supports this significant HRQoL deterioration, with a mean score of 0.75, considerably lower than Italian normative population data [37]. The deficit was evident across all age groups and reached statistical significance among patients aged 65–74 years, consistent with the well-recognized influence of frailty, morbidity, and reduced physiological reserve on post-critical illness recovery in older adults [75]. Notably, male patients exhibited a significantly greater reduction in EQ-5D index value compared with normative references. Several mechanisms may help explain the greater reduction in HRQoL observed among male survivors [76,77]. Men tend to experience a more pronounced loss of skeletal muscle mass during critical illness, which may exacerbate post-ICU weakness and negatively affect long-term mobility trajectories [78]. Additionally, sex-related differences in inflammatory responses, anabolic resistance, and hormonal regulation may impair physical recovery in males to a greater extent than in females [79]. Beyond physiological factors, post-ICU psychological burden may also vary by sex [80]. Studies have shown that men are less likely to report or seek support for anxiety, depression, and post-traumatic stress symptoms following critical illness, which may contribute to poorer HRQoL and reduced engagement in rehabilitation [81,82]. These combined biological and psychosocial factors may help explain the more pronounced HRQoL deficits observed in male survivors of critical illness.

While sex-related differences may influence post-ICU recovery trajectories and contribute to the heterogeneity observed in HRQoL outcomes, it is also important to consider the role of critical illness severity and the intensity of organ support (such as invasive MV). The observed association between the need of MV and lower HRQoL should therefore be interpreted with caution. Rather than indicating a direct causal effect, this relationship likely reflects underlying factors such as the severity of critical illness, prolonged periods of immobility, and ICU-acquired weakness, all of which are known to impair long-term functional recovery [83,84]. These factors not only predispose patients to the development of pressure ulcers, including heel injuries, but also contribute to reduced functional capacity and slower rehabilitation trajectories after ICU discharge, potentially explaining the poorer long-term HRQoL in this subgroup [85].

Collectively, these findings underscore the need for structured, long-term follow-up programs delivered by multidisciplinary teams, including critical care registered nurses, physiotherapists, physicians, and mental health professionals [86]. In addition, the difficulties reported by ICU survivors in resuming daily activities and social participation highlight the relevance of post–intensive care syndrome sequelae, including cognitive and psychological impairments [87]. Future research should assess the cost-effectiveness and feasibility of these interventions across different care settings to inform the development of sustainable post-ICU care pathways and evidence-based nursing practices.

### Strengths and Limitations

There are several strengths to this study. First, it addresses a largely underexplored area of post–intensive care recovery by specifically examining long-term HRQoL and mobility outcomes in ICU survivors with heel pressure ulcer, a subgroup that has received limited attention despite the known impact of pressure ulcers on recovery trajectories. Second, the one-year follow-up achieved a high response rate, enhancing the robustness of the long-term outcome estimates. Third, HRQoL was measured using the EQ-5D-5L questionnaire, a validated and internationally comparable instrument, thus facilitating benchmarking with normative values. Lastly, the study integrates multiple validated tools, such as the Braden Scale, Manchester Mobility Score, and Barthel Index, allowing for a comprehensive assessment of baseline risk factors, functional status, and mobility both during hospitalization and one year after ICU discharge.

Despite these strengths, this study also presents some limitations. Its single-center design may limit external generalizability due to potential variations in clinical practices and pressure ulcer prevention strategies. Moreover, owing to the observational design and the absence of a comparison group without heel pressure ulcers, the findings should be interpreted with caution, as residual confounding cannot be excluded and the independent contribution of heel pressure ulcers to the observed functional and HRQoL outcomes cannot be determined. However, the planned sample size appears appropriate for the study objectives, and enrolling all consecutive eligible survivors over a two-year period reduces selection bias and ensures that the cohort reflects the real-world prevalence of heel pressure ulcers in this ICU.

Another important limitation is the potential for survivor bias. Patients with the most severe clinical conditions or non-healing heel pressure ulcers may have died before ICU discharge or been lost to follow-up. Consequently, the burden of functional impairment and reduced HRQoL reported in this study may underestimate the true impact of heel pressure ulcers on the broader population of critically ill patients. Finally, despite prospective enrollment, some relevant variables (such as detailed rehabilitation exposure, nutritional markers, and wound-specific care during follow-up) were not collected, which may have influenced the interpretation of long-term functional and HRQoL outcomes.

## 5. Conclusions

In conclusion, this study highlights that survivors of critical illness who develop heel pressure ulcers experience persistent impairments in mobility and HRQoL one year after ICU discharge. Invasive MV and prolonged immobility appear to play a key role in shaping long-term outcomes, underscoring the importance of early mobilization and structured rehabilitation pathways. These findings also emphasize the need for proactive prevention strategies and comprehensive follow-up programs aimed at minimizing disability, supporting functional recovery, and ultimately improving the lived experience of ICU survivors affected by heel pressure ulcers.

## Figures and Tables

**Table 1:** Sociodemographic and clinical characteristics.

Variables	n = 52	(%)
Sex (male)	37	71.2%
Presence of caregiver	46	88.5%
Employment status		
Retired	25	48.1%
Unemployed	16	30.8%
Employed	11	21.1%
Comorbidities		
High blood pressure	21	40.4%
Diabetes mellitus	13	25.0%
Chronic obstructive pulmonary disease	12	23.1%
Previous myocardial infarction	10	19.2%
Peripheral vascular disease	7	13.5%
ICU treatments		
Vasoactive drugs	42	80.8%
Mechanical ventilation	36	69.2%
Tracheostomy	18	34.6%
Extracorporeal support	13	25.0%
Pressure ulcers characteristics		
Heel pressure ulcer (before ICU admission)	16	30.8%
Left heel pressure ulcer	13	25.0%
Right heel pressure ulcer	19	36.5%
Bilateral heel pressure ulcer	20	38.5%
Hospital discharge setting		
Rehabilitation facility	23	44.3%
Home	19	36.5%
Other hospital	10	19.2%

Data are expressed as mean (SD) or absolute frequency (%). *Abbreviations:* ICU: Intensive Care Unit.

**Table 2:** Functional and mobility levels.

Variables	n = 52	(%)
Manchester Mobility Score		
1—Passive movements, active exercise, chair position in bed	15	28.8%
2—Site on edge of bed	5	9.6%
3—Hoisted to chair (included standing hoist)	17	32.7%
4—Standing practice	4	7.7%
5—Transfers with assistance	8	15.4%
6—Mobilizing with or without assistance	3	5.8%
7—Mobilizing > 30 min	0	0%
Barthel Index		
Total dependence (0–20 points)	35	67.3%
Severe dependence (21–60 points)	17	32.7%

**Table 3:** EQ-5D-5L frequencies reported by dimension and level.

Level	Mobility	Self-Care	Usual Activities	Pain/ Discomfort	Anxiety/ Depression
Level 1 (no problem)	27 (57.4%)	31 (66.0%)	21 (44.7%)	31 (66.0%)	37 (78.7%)
Level 2 (slight problems)	5 (10.6%)	4 (8.5%)	2 (4.3%)	5 (10.6%)	4 (8.5%)
Level 3 (moderate problems)	8 (17.0%)	6 (12.8%)	12 (25.5%)	9 (19.1%)	5 (10.6%)
Level 4 (severe problems)	6 (12.8%)	4 (8.5%)	5 (10.6%)	1 (2.1%)	1 (2.1%)
Level 5 (extreme problems)	1 (2.1%)	2 (4.3%)	7 (14.9%)	1 (2.1%)	0 (0%)

**Table 4:** Subgroup analysis of EQ-5D index value according to patient characteristics.

Variable		n (%)	EQ-5D Index Value	*p*-Value
Sex	Female	13 (27.7%)	0.842 (0.176)	0.230
	Male	34 (72.3%)	0.712 (0.301)	
Age (years)	<75	38 (80.9%)	0.754 (0.243)	0.493
	≥75	9 (19.1%)	0.748 (0.286)	
BMI (kg/m^2^)	<25	25 (53.2%)	0.765 (0.239)	0.483
	≥25	22 (46.8%)	0.726 (0.329)	
Mechanical ventilation	Yes	33 (70.2%)	0.705 (0.293)	0.037
	No	14 (29.8%)	0.861 (0.196)	
ICU LOS (hours)	<72	10 (21.3%)	0.833 (0.219)	0.119
	≥72	37 (78.7%)	0.724 (0.289)	
Discharge setting	Home	19 (40.4%)	0.827 (0.198)	0.127
	Other	28 (59.6%)	0.682 (0.318)	

EQ-5D value is reported as mean and standard deviation. *Abbreviations:* ICU: intensive care unit; LOS: length of stay; BMI: body mass index.

## Data Availability

The raw data supporting the conclusions of this article will be made available by the authors on request.

## Public Involvement Statement

There was no public involvement in any aspect of this research.

## Guidelines and Standards Statement

This manuscript was drafted in accordance with the STROBE (Strengthening the Reporting of Observational Studies in Epidemiology) guidelines.
